# The Application Method of Nanobubble Conveyor on the Effect of Preventive Oral Hygiene

**DOI:** 10.1155/2020/8871849

**Published:** 2020-10-30

**Authors:** Pei-Ju Lin

**Affiliations:** Department of Commercial Design, National Taichung University of Science and Technology, Taichung 404, Taiwan

## Abstract

There are various methods to generate nanobubbles, and in this study, we experimented using a nanobubble generator with a high-density of stainless steel mesh nozzle to deliver nanobubble water (normal water and two kinds of mouthwash) stream through a tooth tray to clean bacteria coated on the denture. It showed that with various combinations of motor speed settings and pore diameters, a clearing rate of 95% or more could be achieved, while in some combinations, a clearing rate of 100% was possible. This confirmed the plaque removing the function of the nanobubble water streams. The motor speed setting of the nanobubble generator directly influenced the flow velocity and nanobubble diameter of the water stream. However, the nanobubble dimensions were found to have a significant impact on plaque removal. The bubble diameters and plaque removal efficacy were as follows: the smaller the diameter, the slower the flow velocity and the better the plaque removal. The nanobubble formation of mouthwash was better on plaque removal, compared with the soaking method. From these results, we theorized that plaque removal is influenced by the dimension of nanobubbles; smaller bubble diameter led to improved plaque removal efficacy.

## 1. Introduction

The oral cavity is closely related to the tooth structure, but it is difficult to take care of permanent teeth; after the permanent teeth getting worse, many people choose to have replaced dentures. During the replacement process, there will be material and technical issues, and compared to the original permanent teeth in the oral cavity, there will be differences in the performance of dentures or teeth after endodontic treatment [1]. The study [2] expressed a way to optimize the stress distribution of teeth after endodontic treatment. In terms of its characteristics, it effectively proposes a technical solution to dissipate pressure. The pressure to the teeth is related to the oral cleaning and the pressure that the teeth can withstand when the teeth are cleaned. In view of this, this study first used the form of dentures as samples in this experiment.

Differences in personal hygiene and dietary habits also contributed to the many different bacteria strains in the oral cavity. The Human Oral Microbiome Database (HOMD, http://www.homd.org) shows that the current information on the dental plaque has listed more than 25,000 bacteria strains in the oral cavity [3], and up to 10,000 different bacteria may exist in the mouth of just one individual [4]. This often resulted in increased difficulty in maintaining dental hygiene. Teeth are the part of the mouth where most bacteria are found, as they easily form biomembranes over the surface of the teeth. When acidic materials began to erode the enamel, the teeth lose the ability of self-protection. The bacteria on the surface of the teeth and mucosa grow on the nutrients from the saliva; sufficient nutrients will result in elevated bacteria on the dental surface, forming dental plaques and hampering dental and gingival hygiene. The surface of the tooth bacteria grows by saliva nutrients, like amino acids, protein, lipid, carbohydrate, and some inorganics, etc. When the bacteria are sufficiently nourished, they flourish on the surface of the tooth and form dental plaques [5]. Oral hygiene is maintained through the removal of these plaques. Relevant studies point out that patients with chronic diseases need to pay more attention to oral health. The risk of periodontitis in diabetic patients can be twice to three times that of ordinary people, and those with type 2 diabetes are more obvious [6, 7]. Studies have shown that for patients with cardiovascular diseases and diabetes, the pathogens from the periodontal disease will destroy blood vessel walls in the circulation, causing cardiovascular inflammation of the vessel wall, blockage, or severe heart attack [8].

Onaga has also explored using ultrasonic frequency to produce microbubbles and remove dental plaques from a flat surface. Microscopic observation showed that such methods could achieve effective plaque removal, but since the dental structures of the oral cavity are complex, this study did not propose a viable solution for cleaning real teeth or dentures [9]. As there are many ways to produce microbubbles, we propose a method that is of lower cost and easy to implement. We used a motor to blend water (including 2 kinds of mouthwash, Day and Night, and Listerine) and air to generate nanobubbles through a high density of stainless steel mesh nozzle and channel the bubble stream to a specially designed tooth tray to clean the denture inside the tray. This study used the 2 kinds of mouthwash and normal water in a soaking way and nanobubble formation. We aimed to investigate whether nanobubbles generated by the device are able to clean the plaques from the denture and to know the different types of water (including 2 different mouthwash brands) in soaking way and nanobubble formation and whether they have different effects on plaque removal.

## 2. Literary Review

The actions of teeth and tongue are to crush food and transfer it to the digestive organs. Unhealthy teeth will result in less optimal oral cavity function and a higher burden on the gastrointestinal system if the food cannot be properly chewed, affecting body health [10, 11]. In the book *Fundamentals of Oral Histology and Physiology*, Hand and Frank pointed out that dental disease includes cavities, calculus, gingivitis, periodontal disease, and malocclusion, and a large proportion of those related to dental plaque indicated that dental plaque is a composite biomembrane formed by bacteria residing on the surface of teeth and soft tissues. About 2/3 of dental plaque will produce acidic substances that will erode the enamel layer on the surface of the teeth, causing dental cavities [5]. With the advances in molecular biotechnology, researchers have discovered that many of the new oral bacterial species that have yet to be cultured experimentally are related to periodontal disease, demonstrating the biodiversity of oral nanoorganisms [3, 12].

In the plaque cleaning study, in order to understand the difference between the use of electric toothbrushes and manual toothbrushes to clean plaque, subjects were tested and Turesky was used to score plaque before and after brushing [13–15]. Kreifeldt collaborated with DuPont and produced an ergonomically designed toothbrush, REACH. During their study, plaque indicators were applied to the teeth of the subjects to assess the cleaning effects of using different toothbrushes. Plaque removal on different tooth locations with different brushes was professionally assessed and recorded by qualified dentists [16]. Most of the research related to the removal of dental plaque is observed using the dental plaque index. Although this index can explain the cleanliness subjectively, it is difficult to understand this method in the observation of bacteria. Therefore, in order to have a clearer understanding of the effect of removing plaque bacteria, the author uses microbial verification as the basis for quantifying the cleaning effect. Moreover, Lin's study only conducted bacterial estimation on the bottom side of the teeth, not the adjacent faces or the inner and outer faces [17]. So, in the referencing study by Lee et al. [18], we stained the samples in a bacterial solution and then conducted cleaning using various methods.

Bubbles are formed in many natural processes. When gases and liquids are combined under pressure, bubbles of various sizes and shapes are formed. Bubbles can also be formed by emitting electricity, depressurization, increased temperature, ultrasound, and electrolysis of various liquids. The bubble formation process, after multiple splitting, will result in very small dimensions, forming the so-called nanobubbles. Based on the process of air disintegration in water, Fujikawaa developed an instrument that channels air through a compressor into a plate drilled with multiple holes and controlled the rotation of the plate with a motor to generate nanobubbles [19]. In this method, bubbles are cut down by shear force, and the faster the rotation speed, the smaller the diameter of the generated bubbles. Common applications of microbubbles are as follows. (1) Cleaning action: the detergent solution is absorbed around the bubbles to increase the contact surface areas between the detergent and dirt, which improves the cleaning action. Miyamoto performed a study on using microbubbles with an average diameter of 70 micrometers to remove oil residues on surfaces, which had higher cleaning action than normal bubbles; if combined detergent with microbubbles can make detergent be absorbed around bubbles, the cleaning effect is more significant [20]. (2) Sterilization: the sterilization action of ozone can be improved by forming microbubbles and can enhance the sterilization effect; lkeura has used water with ozone microbubbles to remove pesticide residues and pests from vegetables and fruits [21]. Compared with ordinary bubbles, nanobubbles have a smaller diameter and high stagnation in liquids, which can increase gas solubility. Nanobubbles with a small diameter will accumulate in the boundary layer, and the increase in the contact surface area will have a better sterilization effect, and before the collapse, the ion density in the gas-liquid interface is high, which can generate free radical [22].

Based on literature findings, the authors of this study have hypothesized that the size of microbubble is influential on cleaning efficacy (flotation), when used to maintain dental hygiene. The smaller the bubble diameters, the better the cleaning efficacy. In the present study, microbubbles are generated by dissolving air in water.

## 3. Method

In the author's prior study “A Method to Output Microbubbles for Oral Hygiene,” experiments showed the cleansing effect from microbubbles by the MD20 as a sample with the electric motor (13000 rpm) and CNC nozzles [17]. The author has speculated that if the number of nozzle holes is more and the size is smaller, that could be more effective on plaque removal. This study changed the method of making holes on nozzles as using the high density of stainless steel mess supersede CNC. The following experiments were conducted to investigate the influence of the various combinations of variables on denture plaque removal and to see which combination yielded the most optimal removal efficiency. The criteria investigated included various combinations of water flow volume, velocity, and nanobubble dimensions, produced from different motor speed settings with a high density of stainless steel mesh nozzle of the nanobubble generator.This study used the 2 kinds of mouthwash and normal water in a soaking way and nanobubble formation.

### 3.1. Nanobubble Generator and Control Variables

**Figure 1:**
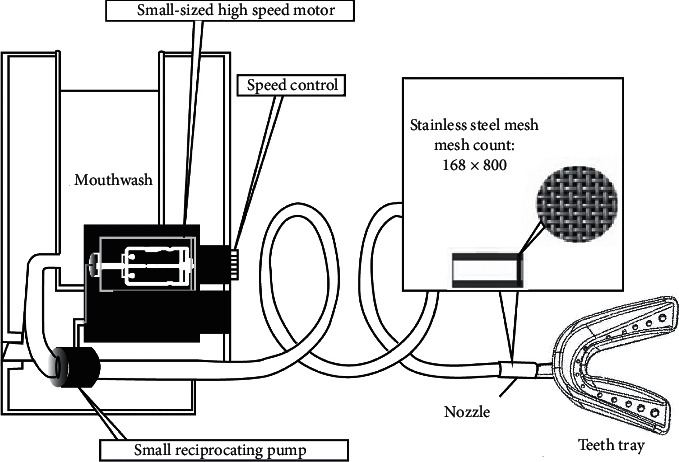
The modified nanobubble generator and the connected teeth tray made from silicone.

Figure 1 shows the small device which used the brushed DC electric motor with 30500 rpm (Voltage 7.2 V, 30 W, Size 27.2∗57 mm), Model Number 380, from Guangdong company, used as a testing device. The processing and assembly were made by TAIWAN PRECISION TRANSMISSION, INC. It also referred to commercially mouth-washing device products as Panasonic, Waterpik, Braun, used as the nanobubble generator, which operated on the same principles as Hasegawa's study [23]. A series of thin slits were placed in the water path, and a mixture of air and water is flushed through these slits. Flow speed differential was produced by the difference in the specific density of air and water, which then expels both air and water and forms nanobubbles. Our device, however, differed from the Hasegawa on the internal tubing that produces nanobubbles; in our device, only the nozzle was changed, not the flow path shape or internal angles. The nanobubble generator has a three-step variable speed setting. The rotation speed was measured with a contact tachometer that measured each step up to 10 times, taking the mean as the representing value. The results were 2500 rpm, 3500 rpm, and 5100 rpm. We built a stainless steel nozzle with CNC (Computer Numerical Control) fabrication, which measured 16 mm in length, 6 mm for the outer diameter, and 5 mm for the inner diameter.

The ejection pore was fabricated with the high density of stainless steel mesh (165 × 800, provided by the May Chun Company) nozzle by electric discharge machining. A total of 9 experimental combinations were produced from three rotational speeds, 2 kinds of mouthwash, and normal water, which allowed the generation of water streams with different flow volumes, velocities, and bubble diameters for carrying out dental plaque removal experiments.

### 3.2. Measurement of Intermediate Variables

This research made a comparison with the high-speed photographs taken before the experiment when the bubble generator was not connected to the soft teeth tray. The flow volume and velocity obtained when combined with the soft teeth tray were significantly higher. Our test confirmed that the combination with a soft teeth tray would reduce the burden of water flow and velocity on the oral cavity. In the current study, we measured the flow volume, velocity, and nanobubble diameters produced by the nanobubble generator and set them as intermediate variables under 9 different experimental conditions. The water flow volume was done by measuring the total water volume for 10 seconds with a measuring cup holding the water ejected from the nanobubble generator connected to a soft teeth tray. The flow volume per second was then calculated, and the measurement was repeated 10 times for the average value. The dimensions of the nanobubbles were measured by photos taken with a high-speed camera. A square glass box (20cm × 20cm × 20 cm) was filled with 10 cm of high-pressure RO (*Reverse osmosis*) water at 23ºC (high-pressure water was used to decrease impurities in the water and to lower their impact on nanobubble generation). The nanobubble generator was then connected to the fabricated nozzles and teeth tray and was placed inside the box. The generator was turned on to eject water for 3 seconds. After the jet stream becomes stable, a high-speed camera was used to photograph the jet stream for 1 second (1,000 frames/sec). Of the 1,000 total photos taken, 500 (250^th^ to 750^th^) were played back at slow speed with the Mega Speed AVI Player software. Of those, 10 clearer photographs are then selected to measure the bubble diameters (millimeter, mm), and the average value was taken as the representative value. From the filmed photos, the position of the same bubble in 10 continuous photos was tracked, and its distance was measured to calculate the flow rate of the intermediate variable (M/S). The measurements of the intermediate variables are shown in Table 1.

### 3.3. Preparation of the Experiment and Materials

To quantify the cleaning efficacy of nanobubbles on dental plaques coated on the denture, we cultivated plaque bacteria in this study. Before the experiments started, we collected bacterial strains from the clinical periodontal patients at a dental clinic. During sampling, a sterilized cotton swab was rubbed evenly around the oral cavity of the patient, and the collected samples were immediately placed inside a sterilized test tube. The samples were cultured using Sabouraud dextrose agar medium and were transfected to a liquid medium. The bacterial culture was then placed on an orbital shaker in the incubator and cultured for 48 hours at 37°C at a speed of 180 rpm. In addition, the denture and teeth tray were prepared for the experiment. The denture sample was created by a dental material company using adult teeth mold supplied from a dentist and consisted of 14 false teeth on the upper jaw of a normal adult. The teeth tray envelopes the denture and is connected on one end to the nanobubble generator, which channels the water stream out of the nozzle pore to clean the denture. The tray is made of medical silicone rubber with a hardness of 40. The water ejection ports are fabricated corresponding to each tooth on the denture. There are 14 holes.

### 3.4. Experiment Steps in Dental Plaque Removal

**Figure 2:**
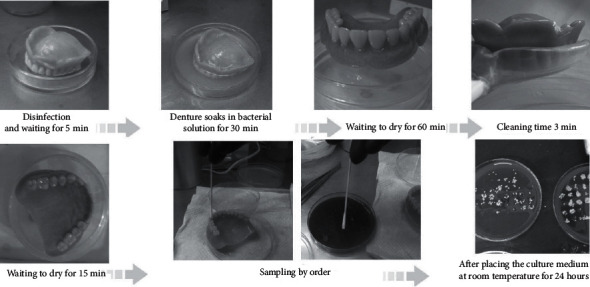
Experimental procedure for cleaning the denture.

The dental plaque removal experiment was conducted in a sterilized laminar flow cabinet. The test denture of the control group was first sterilized and immersed in a 250 ml square container with a solution containing 8 × 10^10^ CFU/ml bacterial solution for 30 minutes, then dried for 60 minutes, and then placed in a sterilized 500 ml container with 100 ml mouthwash (control group is soaking method). After 3 min cleaning is completed, the residual bacteria on the denture were then calculated based on the methods and steps from Lee [18]. Briefly, the cleaned denture is placed in another sterilized glass dish and dried for 30 minutes with the teeth surface facing upward. The dental bacteria on it were sampled by rubbing a 5 mm sterile cotton swap on each tooth surface. There are three faces on each tooth, but the bottom parts of the four incisors and two canines were not sampled because they were relatively small and easier to clean, which meant that small differences in cleaning outcomes were expected. Therefore, a total of 36 tooth surfaces were sampled (6 × 2 + 8 × 3 = 36), with 36 sampling times and 36 cultures, as each collected sample was plated separately. The colony units were then counted with the colony counter. The estimation of bacterial removal in this study was not specific to a single strain, but rather to the total number of bacteria. Since there were many bacterial strains in the oral cavity, a single strain is not representative of the overall situation. Since only one denture was used for each round of the cleaning experiment, the denture was carefully disinfected after each test to make sure that it is completely sterilized and then reimmersed in a bacterial solution for the next test. The experimental group cleaning procedure is shown in Figure 2: after sterilizing the cleaned denture from the previous test, the denture was set for 5 minutes and immersed in the bacterial solution for 30 minutes with the teeth side facing downward. The denture was then dried for 60 minutes, then inserted into the teeth tray with the desired experimental combination, washed for 3 minutes, and then dried again for 30 minutes, and pressed onto the culture medium for 30 seconds for the colony-counting test.

The cleaning experiment was performed according to each combination of variables for a total of 20 times. There are three faces on each tooth, but the bottom parts of the four incisors and two canines were not sampled because they were relatively small and easier to clean, which meant that small differences in cleaning outcomes were expected. After the culture media were incubated for 24 hours, they were tested for the existence of plaque. The percentage of sterile surfaces was used to indicate cleaning efficacy. If 20 out of 36 media displayed no dental bacteria after cleaning, it meant that 20 tooth surfaces were cleaned completely and the cleaning efficacy was 20/36 × 100 = 83.33%. The resulting cleaning efficacy of each experimental combination is shown in 2. Three such groups were formed with eight surfaces each (buccal surface (premolars (PM)-BS)), occlusal surface (PM-OS), and lingual surface (PM-LI) of PM and molars) and two groups with six surfaces each (labial surface (IC-LA) and lingual surface (IC-LI) of incisors and canines). During the experiment course, the temperature of the bacterial console was controlled to prevent temperature changes in bacteria solutions from affecting the overall bacteria solution volume.

## 4. Results

### 4.1. The Effect of Control Variable on Flow Volume

The results of the water flow volume produced from the various combinations of the 2 kinds of mouthwash and normal water and three speed settings of the bubble generator are shown in Table 1. From the data on the effect of control variables on water flow volume, the flow volume seemed to increase with faster motor speed. Using normal water was significantly higher than all the other although there were little differences between the two mouthwash. Table 1 also showed the results of two-way ANOVA, which showed that the motor setting significantly influenced the water flow volume (*P* < 0.01).

### 4.2. The Effect of Control Variable on Water Flow Velocity

In Table 1, the effects of control variables on flow velocity have shown that as motor speed increases, flow velocity increased slightly and was also significantly affected by different types of mouthwash and water. Results from ANOVA also showed that motor speed and different types of water achieved a 0.05 significance level.

### 4.3. The Effect of Control Variable on the Diameter of the Nanobubbles

Table 1 shows that the diameters of nanobubbles decreased slightly with increasing motor speed. The ANOVA results only show that the motor speed settings were significant. Overall, the faster the motor speed settings, the smaller the diameter of the nanobubbles; the slower the motor speed settings, the larger the bubble dimensions.

### 4.4. Correlation between the Control Variables and Plaque Removal

Concerning the effect of control variables on plaque removal (Table 1), the average efficiency was 95% and above, with the mouthwash into the nanobubble having the best efficacy. The effects of different motor speeds on plaque removal were not significant; ANOVA results also showed that only the influence of using different types of water to clean has achieved 0.05 significance. Table 1 also shows that when the motor speeds were fast and using mouthwash, the plaque removal efficacy was optimal and achieved over 100%.

### 4.5. Correlation between Intermediate Variables and Plaque Removal

We then investigated the effect of intermediate variables (water flow volume, flow velocity, and nanobubble dimensions) on plaque removal. We performed backward multiple regression analysis, and the results are shown in Table 2. At a significance level of *α* = 0.05, only the nanobubble diameter has significantly affected plaque removal. The negative regression coefficient value showed that, as the bubble diameter decreases, the plaque removal efficacy was increased. The results from the author's previous single-nozzle study did not show a significant influence of flow volume, velocity, and bubble diameter on plaque removal; however, in the current study, with the three nozzles, we observed the significant influence of bubble diameter on plaque removal, which confirmed our hypothesis.

### 4.6. Plaque Removal of Different Areas in Different Methods

**Figure 3:**
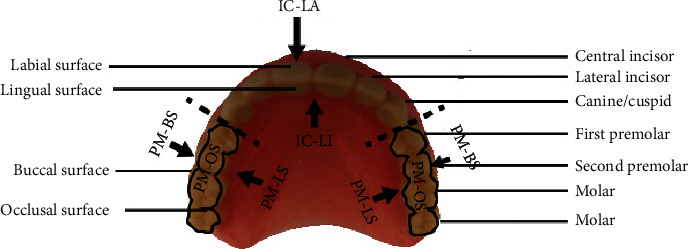
Tooth surface areas for sampling after denture cleaning (premolars (PM), buccal surface (PM-BS), occlusal surface (PM-OS), the lingual surface (PM-LI) of PM and molars, and two groups with six surfaces each (labial surface (IC-LA) and lingual surface (IC-LI) of incisors and canines)).

In addition to an overall analysis of the efficacy in plaque removal, this study also divided the denture into five parts, as shown in Figure 3, which included the buccal (PM-BS), occlusal (PM-OS), and lingual (PM-LI) surfaces of the premolars and molars, and the labial (IC-LA) and lingual (IC-LI) surfaces of the incisors and two canines. As shown in Table 3, a more in-depth analysis was conducted to determine the cleaning efficacy in each area, in terms of the percentage of sterile tooth surfaces. For Areas A, B, and C, namely, the buccal (PM-BS), occlusal (PM-OS), and lingual (PM-LI) surfaces of the premolars and molars, the combinations of experimental conditions that cleaned the most tooth surfaces were A & B brands of mouthwash with a higher rotor speed (5100 rpm). There are less sterile faces by using the soaking method and the least sterile faces by soaking in normal water, only 5 faces, in 13.88%.The two kinds of mouthwashes were more significant on plaque removal in nanobubble formation compared with the soaking method. The Areas D and E, namely, the labial (IC-LA) and lingual (IC-LI) surfaces of the incisors and canines, were unable to clean-up in 5100 rpm speed with normal water.

## 5. Conclusion

In the author's past research, the use of commercially available MD20 fuselage and CNC machined nozzles as the testing machine for producing microbubbles limited the number of holes and the size of the aperture [17]. Moreover, the method of testing tooth bacteria only uses the bottom surface, and it is not possible to fully understand the status of the bacteria removal on each side of the tooth. In this study, we proposed using a nanobubble generator outfitted with a high density of stainless steel mesh nozzle that is connected to a teeth tray to clean dental plaque bacteria on the denture. Our results showed that in all experimental combinations of motor speed settings and three different types of water (including two brands of mouthwash), dental plaque removal efficacy of about an average 95% or more was achieved, and in some combinations up to 100% removal efficacy was achieved, which validated the plaque removal capability of the nanobubbles. Generally, the higher motor speed settings with nanobubble formation of mouthwash resulted in better plaque removal. The motor speed setting of the nanobubble generator directly influenced the flow velocity and nanobubble dimensions of the water stream; at a higher motor setting, the flow velocity increases and the nanobubble dimensions decrease; however, the nanobubble dimensions were found to have a significant impact on plaque removal. The bubble diameters and plaque removal efficacy are as follows: the slower the velocity and the smaller the bubble dimensions, the better the plaque removal. From these results, we theorized that plaque removal is influenced by the dimension of nanobubbles; smaller bubble diameter led to improved plaque removal efficacy, which was also confirmed by our regression analysis.

In summary, to make mouthwash became the nanobubble function could help to clean dental bacteria. In this study, only 2 brands of mouthwash water turn into bubbles. However, due to different oral environment factors, in the present study, a denture was substituted as the test subject instead of a real human oral cavity. These results may be used for future improvements to the design of the microbubble generator for dental hygiene. Future studies may perfect the experiment design to accurately obtain the bacterial removal efficacy of each face of the teeth. Experimental verification of conduct plaque removal by this way of dental washer connects the ejection nozzle to an ergonomically designed teeth tray that fits the tooth configuration of a typical human oral cavity. We hope that the produced microbubbles by this approach can definitely solve the dental hygiene issue for long-term bedridden patients.

## Figures and Tables

**Table 1:** Statistics and ANOVA results regarding the effects of control variable on (A) flow volume, (B) flow velocity, (C) nanobubble diameters, and (D) bacteria removal.

	(A)			(B)			(C)			(D)		
	2500	3500	5100	2500	3500	5100	2500	3500	5100	2500	3500	5100
Normal water	4.2	5.4	6.6	0.06	0.08	0.09	0.0055	0.0049	0.0032	86.11	94.44	97.22
A brand mouthwash	2.6	2.9	3.1	0.03	0.04	0.05	0.0061	0.0055	0.0049	94.44	97.22	97.22
B brand mouthwash	2.5	3.0	3.2	0.03	0.04	0.04	0.0055	0.0052	0.0048	94.44	100	100
Mean	3.1	3.8	4.3	0.04	0.05	0.06	0.0057	0.0052	0.0043	91.66	97.22	98.15

	Source	Sum of squares	d*f*		Mean square		*F*		Sig.		*R* ^2^	

(A) ANOVA	Different types of water	12.669	2		6.334		23.081		0.006		0.930	
	Rotor speed	2.169	2		1.084		3.951		0.113			
	Error	1.098	4		0.274							
	Total	140.630	9									
	Corrected total	15.936	8									

(B) ANOVA	Different types of water	0.003	2		0.001		53.200		0.001		0.970	
	Rotor speed	0.001	2		0.000		11.200		0.023			
	Error	0.000	4		2.778E-5							
	Total	0.027	9									
	Corrected total	0.004	8									

(C) ANOVA	Different types of water	1.447E-6	2		7.233E-7		3.647		0.125		0.850	
	Rotor speed	3.0204E-6	2		1.510E-6		7.613		0.043			
	Error	73933E-7	4		1.983E-7							
	Total	0.000	9									
	Corrected total	5.260E-6	8									

(D) ANOVA	Different types of water	73.769	2		36.884		7.830		0.041		0.870	
	Rotor speed	48.026	2		24.013		5.098		0.079			
	Error	18.842	4		4.711							
	Total	82526.858	9									
	Corrected total	140.637	8									

**Table 2:** Regression analysis on the effect of intermediate variables and dependent variables.

Dependent	Independent	B	Std. Error	Beta	v	Sig.	*R*	*R* ^2^
C	Nanobubble diameter	−6290.191	2246.813	−1.216	−2.800	**0**.**038**	0.817^c^	0.668

**Table 3:** Statistical table of the number of bacteria-free teeth surfaces in the soak method and nanobubble formation.

			A	B	C	D	E	No. of sterile faces	Bacteria removal (percentage)
Soaking method (control group)	Water		2	2	0	0	1	5	13.88
	A brand mouthwash		7	7	6	4	6	30	83.99
	B brand mouthwash		8	7	6	5	6	32	88.89

Nanobubble formation (experimental group)	Water	2500	7	6	6	6	6	31	86.11
		3500	7	8	7	6	6	34	94.44
		5100	7	8	8	5	6	34	94.44
	A brand mouthwash	2500	8	7	7	6	6	34	94.44
		3500	8	8	7	6	6	35	97.22
		5100	8	8	8	6	6	36	100
	B brand mouthwash	2500	8	7	8	6	6	35	97.22
		3500	8	8	7	6	6	35	97.22
		5100	8	8	8	6	6	36	100

## Data Availability

No data were used to support this study.
